# Supplementary material for: Stochastic Gradient Descent-like relaxation is equivalent to Metropolis dynamics in discrete optimization and inference problems
Source: Sci Rep. 2024 May 21;14:11638. doi: 10.1038/s41598-024-62625-8 (PMC11639713; doi:10.1038/s41598-024-62625-8)
Supplement: Supplementary file 1 — Supplementary Information. [file 41598_2024_62625_MOESM1_ESM.pdf]

Supplementary Information for "Stochastic Gradient Descent-like relaxation is equivalent to Metropolis dynamics in discrete optimization and inference problems"

November 10, 2023

1 Computation times

In Fig. 1 we show the required time in seconds to run 1000 sweeps of MC or SGD-like algorithms (with the code available at `https://www.dropbox.com/sh/in2wxrycx6nhznh/AADsYarQaaSYRkgLYYbzNm17a?dl=0`). Remember that a sweep corresponds to the attempt to change the color of N variables. The time for a single sweep grows linearly with the size of the system N. SGD-like algorithm is a bit slower than the MC algorithm because it needs to extract $c \cdot N$ random numbers at each sweep to build the mini-batch (while MC only needs at most N random numbers for each sweep). This part of the code could be optimized, but we leave it for future work, given that running times are anyhow short enough to use both algorithms on large instances.

The runs have been performed on a single CPU, with processor Intel(R) Core(TM) i7-6500U CPU @ 2.50GHz.

2 Additional numerical experiments

In this section, we will show additional numerical evidence to complement the results already illustrated in the main text.

In the main text, we have described the Gradient-Descent (GD)-like algorithm. It finds a solution to the planted coloring problem only for connectivities larger than $c_{GD}(N)$: In the left part of Fig. 2 we show the probability of recovering the planted solution, as a function of the average connectivity of the graph c. We can define $c_{GD}(N)$ as the connectivity at which the recovery probability becomes different from 1. This threshold seems to scale logarithmically with N: $c_{GD}(N) \simeq A \log(N)$, with $A = O(1)$. In fact in the right part of Fig. 2 we show the probability to recover the planted solution as a function of $c/\log(N)$. When plotted as a function of this rescaled parameter, the points at which the

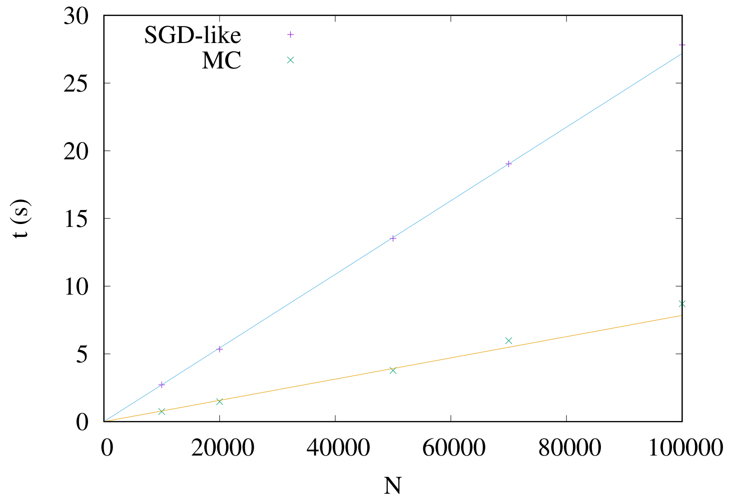

Figure 1: Required time in seconds to run 1000 sweeps of MC or SGD-like algorithms as a function of the size of the system. The continuous lines are the best linear fits.

recovery probability becomes different from 1 collapse for different N, indicating that the connectivity at which the recovery fails scales as $c_{GD}(N) \simeq A\log(N)$. The threshold for the GD-like algorithm thus diverges in the large N limit.

In the main text, we have then introduced two algorithms, the MC and the SGD-like algorithm, for which stochasticity helps to find the signal. We call nucleation time the time at which the signal is nucleated. Comparing the MC and SGD-like algorithm at values of the parameter T and B such as to have the same plateau energy, as in Fig. 2 of the main text, we have shown in Fig. 3 of the main text that also the average nucleation time and its standard deviation above different samples match for the two algorithms. Here, in Fig. 3 we show that indeed the whole distribution of the nucleation times coincides for the two algorithms at a fixed size N of the graphs.

In the main text, we have shown that there is an equivalence between the MC and the SGD-like algorithms when performing an inference task. However, this equivalence also works if one wants to solve the optimization problem of finding a good coloring configuration in a problem without the planted solution. The model we have studied is always the same — the planted 5-coloring problem on random graphs — but for low enough connectivities ($c < c_{MC} = 18$) the inference task is impossible to solve and thus the algorithms are performing an optimization task over random problems. In Fig. 4, we show that also for the optimization problem, the two algorithms behave quantitatively in the same way. At high T (low B) the two algorithms reach a paramagnetic state while at low T (high B) the two algorithms enter glassy states and the dynamics is an out-equilibrium one.

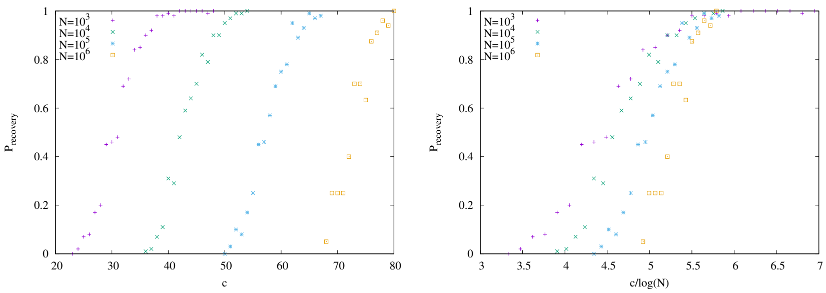

Figure 2: **Left**: Probability to recover the planted solution with a Monte Carlo algorithm at zero temperature, that is a gradient-descent-like algorithm, as a function of the average connectivity of the graph c. The recovery probability changes by changing the size of the system N and seems to go to 0 in the thermodynamic limit for any finite connectivity. **Right**: Probability to recover the planted solution with a GD-like algorithm, as a function of $c/\log(N)$. The points at which the recovery probability becomes different from 1 collapse for different N, indicating that the connectivity at which the recovery fails scales as $\log(N)$ and thus diverges in the thermodynamic limit.

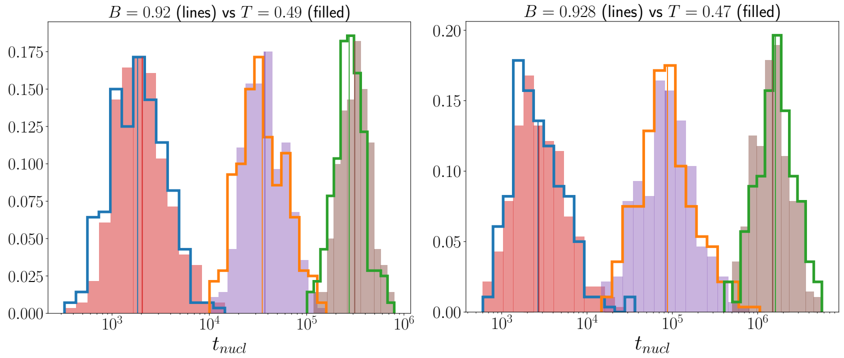

Figure 3: Histogram of nucleation times for the MC algorithm at temperature T (filled curves) and for SGD-like algorithm at mini-batch size B (lines), at the same values of T and B extracted in Fig. 2 of the main text. Three different sizes are considered in each panel from left to right, $N = 10^3, 10^4$, and 10^5 and the distribution is extracted from 280 different samples. Vertical lines show the average nucleation time for each case (the ones shown in Fig. 3 of the main text).

We also underline that the fact that the detailed balance (eq. 5 in the main text) is not fully satisfied is consistent with the numerical findings of figure 4, which shows that the effective value of B for $T = 0.4$ changes slightly from $B = 0.95$ to $B = 0.94$ when going from $c = 19$ to $c = 15$. If eq.5 in the main

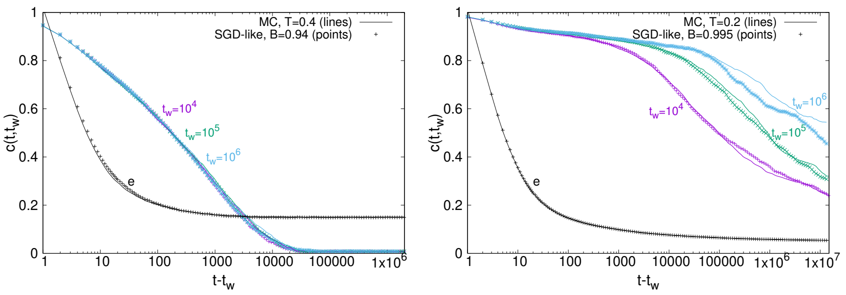

Figure 4: Same as figure 4 of the main text but for $c = 15$, single system of size $N = 10^5$. In this case, for $c = 15$, there is no temperature interval between the paramagnetic and aging regimes where the planted state can be retrieved. At high T (low B) the two algorithms reach a paramagnetic state while at low T (high B) the two algorithms enter glassy states and the dynamics is an out-equilibrium one.

text was to be satisfied, then the resulting expression for G would imply a $T(B)$ relation which is independent of c. Here we can explain the observed c dependence as coming from the fact that increasing c also the number of curves $G(B, s, u)$ increases (we have more choices for s and u), and thus the effective $T(B)$ relation (that we modeled with the arithmetic average of the curves) will reasonably slightly vary with c.
